# Real-World Efficacy and Safety of Atezolizumab for Advanced Non-Small Cell Lung Cancer in Japan: A Retrospective Multicenter Analysis

**DOI:** 10.3390/jcm13247815

**Published:** 2024-12-20

**Authors:** Masahiko Okada, Keiko Ohgino, Kohei Horiuchi, Koichi Sayama, Daisuke Arai, Mayuko Watase, Keigo Kobayashi, Takeshi Terashima, Kota Ishioka, Masayoshi Miyawaki, Fumio Sakamaki, Keita Masuzawa, Hideki Terai, Hiroyuki Yasuda, Kenzo Soejima, Koichi Fukunaga

**Affiliations:** 1 Division of Pulmonary Medicine, Department of Medicine, Keio University School of Medicine, 35 Shinanomachi, Shinjuku-ku 160-0016, Tokyo, Japan; mokada332@gmail.com (M.O.); koheihoriuchi17@gmail.com (K.H.); hidekit926@gmail.com (H.T.); hiroyukiyasuda@a8.keio.jp (H.Y.); ksoejima@cpnet.med.keio.ac.jp (K.S.); kfukunaga@keio.jp (K.F.); 2 Icahn School of Medicine at Mount Sinai, Mount Sinai Morningside and West, 1000 Tenth Avenue, New York, NY 10019, USA; 3 Kawasaki Municipal Hospital, 12-1 Shinkawadori, Kawasaki-ku 210-0013, Kanagawa, Japan; ksayama@kmh.gr.jp; 4 Pulmonary Division, Department Internal Medicine, Saiseikai Utsunomiya Hospital, 911-1 Takebayashimachi, Utsunomiya 321-0974, Tochigi, Japan; dai-ara2008@kdr.biglobe.ne.jp; 5 Department of Respiratory Medicine, National Hospital Organization Tokyo Medical Center, 2-5-1 Higashigaoka, Meguro-ku 152-8902, Tokyo, Japan; mayukokoshibe@gmail.com; 6 Department of Medicine, Sano Kousei General Hospital, 1728 Horigomecho, Sano 327-8511, Tochigi, Japan; keigokbys@gmail.com; 7 Tokyo Dental College, Ichikawa General Hospital, 5-11-13 Sugano, Ichikawa-shi 272-0824, Chiba, Japan; terasima@tdc.ac.jp; 8 Department of Medicine, Tokyo Saiseikai Central Hospital, 1-4-17 Mita, Minato-ku 108-0073, Tokyo, Japan; kotaishioka@gmail.com; 9 Department of Pulmonary Medicine, Eiju General Hospital, 2-23-16 Higashiueno, Taito-ku 110-8645, Tokyo, Japan; mmiyawaki1221@hotmail.com; 10 Tokai University Hachioji Hospital, 1838 Ishikawa-cho, Hachioji-shi 192-0032, Tokyo, Japan; fsakamak@tokai-u.jp; 11 Department of Respiratory Medicine, Kitasato University Kitasato Institute Hospital, 5-9-1 Shirokane, Minato-ku 108-8642, Tokyo, Japan; kmasu85@gmail.com; 12 Keio Cancer Center, Keio University School of Medicine, 35 Shinanomachi, Shinjuku-ku 160-0016, Tokyo, Japan

**Keywords:** immunotherapy, monotherapy, adverse events, elderly patients, performance status

## Abstract

**Background/Objectives:** Atezolizumab, an immune checkpoint inhibitor (ICI), was used in a phase III clinical trial, i.e., the OAK trial, of previously treated patients with non-small cell lung cancer. We aimed to evaluate the real-world efficacy and safety of atezolizumab in a non-selected population and identify the clinical characteristics that influence its efficacy. **Methods:** This was a multicenter, retrospective, single-arm observational study. Seventy-four patients with advanced non-small cell lung cancer, who received atezolizumab monotherapy at Keio University and affiliated hospitals in Japan between April 2018 and March 2019, were enrolled. The follow-up period was until 28 February 2024. The efficacy of treatment and adverse events were reviewed retrospectively. Statistical analyses using Pearson’s χ^2^ test, Fisher’s exact test, log-rank test, and Student’s *t*-test were performed. **Results:** The median age of patients was 70 (range, 45–85) years. The overall survival duration was 7.54 (95% confidence interval [CI], 5.14–11.3) months, and the median time to treatment failure (TTF) was 2.00 (95% CI, 1.75–2.54) months. Patients treated with atezolizumab as their first ICI had a longer TTF than those treated with atezolizumab as their second or subsequent ICI (*p* = 0.04). **Conclusions:** Atezolizumab may be more effective when used as the first ICI for previously treated patients and may be safely used in elderly patients with non-small cell lung cancer in real-world settings.

## 1. Introduction

Lung cancer is the foremost contributor to cancer-related deaths worldwide [1]. Chemotherapy is the primary treatment for advanced cancer; however, several patients develop resistance to conventional cytotoxic agents. Immune checkpoint inhibitors (ICIs), such as nivolumab, pembrolizumab, and atezolizumab, have significantly improved the prognosis of patients with advanced lung cancer [2,3,4]. In Japan, nivolumab and pembrolizumab were approved in 2015 and 2016, respectively, for unresectable advanced non-small cell lung cancer (NSCLC), whereas atezolizumab was approved in 2018. We previously reported the real-world efficacy and safety data for nivolumab [5]. Nivolumab and pembrolizumab are monoclonal antibodies targeting programmed cell death protein 1 (PD-1), whereas atezolizumab is a monoclonal antibody targeting programmed death-ligand 1 (PD-L1). Unlike anti-PD-1 agents, anti-PD-L1 therapy such as atezolizumab may lead to a distinct profile of immune-related adverse events (irAEs) [6], which warrants careful evaluation in specific populations, such as elderly patients. The POPLAR and OAK studies included patients who had previously received one or two cytotoxic chemotherapies. Therefore, data on the safety and efficacy of atezolizumab in patients who had previously received three or more chemotherapies, including ICIs, are scarce [7,8]. The J-TAIL study, conducted in Japan, reported real-world data in 2023, but the reasons for the discontinuation of ICIs prior to atezolizumab were not reported [9]. Real-world data provide a critical perspective, bridging the gap between clinical trials and daily practice. Unlike the tightly controlled clinical trials, the real-world studies reflect the diversity of everyday clinical settings, including patients with multiple prior therapies and those with complex medical histories. Elderly patients, who represent a significant proportion of lung cancer cases, are often underrepresented in clinical trials. This demographic faces unique challenges, including comorbidities and reduced physiological reserve, which may increase their vulnerability to adverse events. Although previous studies suggest that irAE rates in elderly patients may be comparable to those in the general population [10], evidence remains limited, necessitating further research in this area. Addressing these gaps is especially relevant in Japan, where the aging population demands evidence-based care tailored to elderly patients. This study evaluated the reasons for the discontinuation of ICIs prior to atezolizumab. In this study, we, the Keio Lung Oncology Group (KLOG), included medium-sized hospitals with 300 beds and large hospitals with 1000 beds in the Kanto area that we believe are reflective of real-world settings. We aimed to evaluate the real-world efficacy and safety of atezolizumab in a non-selected population of patients who had previously received ICIs. Our study aimed to assess the real-world efficacy and safety of atezolizumab in an unselected population of patients in Japan who had prior exposure to ICIs. Furthermore, we focused on evaluating the safety and tolerability of atezolizumab in elderly patients, addressing a critical gap in evidence for this subgroup.

## 2. Materials and Methods

### 2.1. Study Population

Seventy-four previously treated patients with advanced NSCLC who received atezolizumab at Keio University and nine affiliated hospitals (KLOG) between April 2018 and March 2019 were enrolled. Patients diagnosed with NSCLC based on histology or cytology, who showed disease progression during or following their initial treatment, were deemed eligible. With approval from the ethics committee, patients who received atezolizumab during the applicable period were identified at each facility. The study was disclosed to patients as an opt-out, and cases were reviewed and extracted by the responsible personnel at each facility. In addition to patients with stage IIIB–IVB disease and postoperative recurrence, those with stage IIA or IIB disease who chose chemotherapy due to personal preference or other considerations were also included. Patients with stage IIIA disease who met the following criteria were included: (1) diagnosis of nonresectable or progressive disease (PD) after initial chemotherapy and (2) concomitant radiotherapy. Information on clinical characteristics, prior treatments, and outcomes was collected from the patients’ medical records. Patients received 1200 mg of intravenous atezolizumab every 3 weeks (licensed dose and administration method in Japan). This study and the use of the opt-out method were approved by the ethical review board of Keio University and its affiliated hospitals. Data were accumulated until 28 February 2024. This study was conducted in accordance with the 1964 Declaration of Helsinki, as revised in 2013.

### 2.2. Target Lesion Assessment

The baseline dimensions of the target lesions were assessed prior to the initiation of atezolizumab treatment using imaging techniques, including chest radiography, computed tomography (CT), and magnetic resonance imaging (MRI). The clinical response to atezolizumab was evaluated using the same imaging methods in conjunction with the Response Evaluation Criteria in Solid Tumors (RECIST), version 1.1 [11]. Tumor responses to atezolizumab were categorized as follows: complete response (CR), indicating the disappearance of all target lesions; partial response (PR), defined as a reduction of ≥ 30.0% in the sum of the diameters of the target lesions; progressive disease (PD), characterized by an increase of ≥ 20.0% in the sum of the diameters of the target lesions; and stable disease (SD), representing changes insufficient to meet the criteria for PR or PD. In cases where measurable disease was absent, PD was defined by unequivocal progression. Adverse events (AEs) associated with atezolizumab were documented based on the Common Terminology Criteria for Adverse Events (CTCAE), version 4.0. Overall survival (OS) was calculated as the time from the first administration of atezolizumab to death from any cause.

### 2.3. Clinical Characteristics

The analyses included the following clinical characteristics: age, sex, disease stage, Eastern Cooperative Oncology Group (ECOG) performance status (PS), histological subtype, epidermal growth factor receptor (*EGFR*)/anaplastic lymphoma kinase (*ALK*) mutation status, presence of metastases, smoking status, number of previous lines of treatment, previous irradiation, previous ICI treatment, and PD-L1 expression (tumor proportion score [TPS]) expression.

### 2.4. Statistical Analyses

Statistical analyses were conducted using Pearson’s χ^2^ test, Fisher’s exact test, log-rank test, and Student’s *t*-test to assess the clinical characteristics associated with atezolizumab efficacy. A two-sided *p*-value of <0.05 was considered statistically significant for all tests. Variables showing a significant association with a *p*-value < 0.05 were included in the stratification analysis using the Cochran–Mantel–Haenszel test. All statistical analyses were performed using R (R Core Team [2022]), R: A Language and Environment for Statistical Computing (R Foundation for Statistical Computing, Vienna, Austria), and JMP software version 16.2.0 (SAS Institute, Cary, NC, USA). Data visualization was also carried out using R.

## 3. Results

### 3.1. Clinical Characteristics of the Study Population

A total of 74 patients with stage IIA–IVB disease or postoperative recurrence were included in this study. Table 1 provides a summary of their baseline clinical characteristics. The median age of the patients was 70 years (range: 45–85), with 18 patients (24.3%) aged 75 years or older. Among the 74 participants, 56 (75.7%) were male, and 18 (24.3%) were female. The median age was 70 (range, 45–85) years, with 18 (24.3%) patients aged ≥ 75 years. Of the 74 patients, 56 (75.7%) were men and 18 (24.3%) were women. The ECOG PS scores were 0 in 38 (51.3%) patients, 1 in 31 (41.9%), 2 in 3 (4.1%), and 3 in 2 (2.7%). None of the patients had an ECOG PS score of 4. The histological subtypes were as follows: adenocarcinoma in 56 (75.7%) patients, squamous cell carcinoma in 6 (8.1%), and others in 12 (16.2%). Nine (12.2%) patients had an EGFR mutation-positive status. None of the patients tested ALK mutation-positive. Ten (13.5%) patients had central nervous system (CNS) metastases at baseline. Twenty-one (28.4%) patients underwent brain irradiation. Of the 74 patients, 53 (71.6%) were current or ex-smokers and 17 (23.0%) were nonsmokers. In addition, 29 (39.2%) and 45 (60.8%) patients were treated with atezolizumab as second-line treatment and third-line (or higher) treatment, respectively. One patient received atezolizumab as the tenth-line treatment, three as the eighth-line treatment, four as the seventh-line treatment, and six as the sixth-line treatment. Twenty-five (33.8%) patients had previously undergone chest radiotherapy. Our study included patients with the following comorbidities: diabetes (12, 16.2%), thyroid disease (2, 2.7%), interstitial pneumonitis ([IP], 4, 5.4%), and chronic renal disorders (4, 5.4%). Moreover, seven patients had a history of another malignant disease: breast cancer (*n* = 1), bladder cancer (*n* = 1), basal cell carcinoma (*n* = 1), malignant lymphoma (*n* = 1), gastric and esophageal cancer (*n* = 1), esophageal and gingival cancer (*n* = 1), and submandibular gland cancer (*n* = 1).

### 3.2. Objective Clinical Responses and Time to Treatment Failure (TTF)

**Figure 1:**
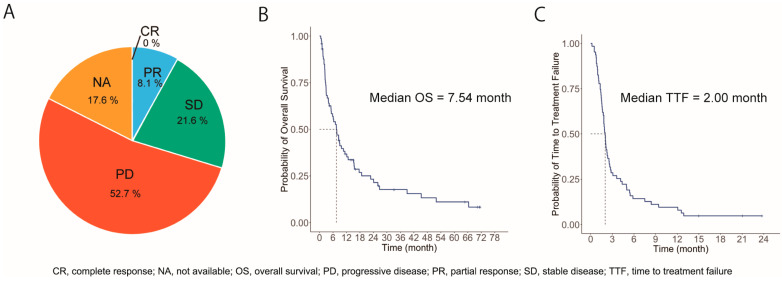
Overall response, overall survival, and progression-free survival of all 74 patients (**A**) Clinical response to atezolizumab (**B**) Kaplan–Meier plot of overall survival (**C**) Kaplan–Meier plot of progression-free survival.

The RECIST criteria were used to assess for treatment efficacy in 49 patients; the remaining 25 patients could not be evaluated for treatment efficacy using these criteria owing to reasons such as lesions that could not be evaluated, including pleural effusions (*n* = 11), treatment discontinuation before the evaluation of treatment efficacy (*n* = 12), or data not collected (*n* = 2). The objective response rate was 8.1%, with the inclusion of all PRs, and the disease control rate was 29.7% (Figure 1A). The median OS duration was 7.54 (95% confidence interval [CI], 5.14–11.3) months for all patients (Figure 1B). Moreover, the median TTF duration was 2.00 (95% CI, 1.75–2.54) months for all patients (Figure 1C). Notably, five cases (6.8%) achieved long-term survival of more than five years from the start of administration.

### 3.3. Clinical Characteristics and Treatment Responses

**Figure 2:**
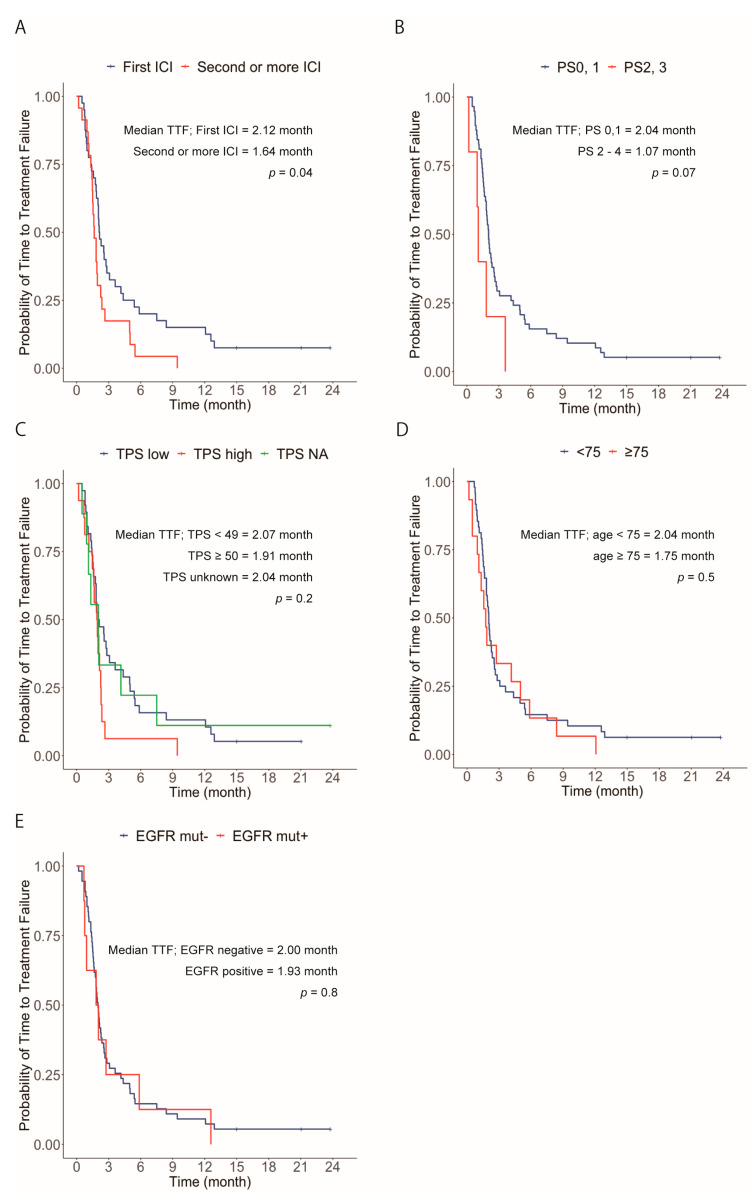
Progression-free survival analyzed by various factors. (**A**) Kaplan–Meier plot of patients for whom atezolizumab was the first ICI and for those who received second or more ICIs (**B**) Kaplan–Meier plot of patients with a PS of 0 and 1 and 2 and 3 (**C**) Kaplan–Meier plot of patients with PD-L1 TPS < 49, PD-L1 TPS ≥ 50, and NA PD-L1 TPS (**D**) Kaplan–Meier plot of patients aged ≥ 75 and < 75 years (**E**) Kaplan–Meier plot of patients with EGFR mutation-positive and EGFR mutation-negative statuses. Abbreviations: ICI, immune checkpoint inhibitor; mut, mutation; NA, not available; PS, performance status.

**Figure 3:**
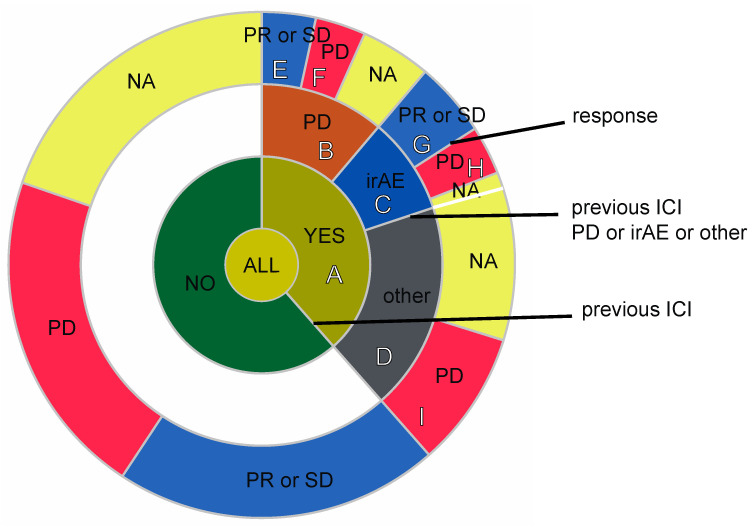
Patients classified based on whether they had been treated with a previous ICI, the reason for the change from a previous ICI was PD or irAE or otherwise, and their best response to atezolizumab. Letters (**A**–**I**) indicate the respective groups. Abbreviations: ICI, immune checkpoint inhibitor; mut, mutation; NA, not available; PD, progressive disease; PR, partial response; SD, stable disease.

Differences in age, sex, ECOG PS score, histological subtype, presence or absence of CNS metastases, smoking status, number of previous lines of treatment, and PD-L1 (TPS) expression were not significantly associated with response to atezolizumab (Table 2). A multivariate analysis was performed using these variables; however, no significant factors were identified. In addition, the differences in response to atezolizumab between those with and without metastasis were not statistically significant (Supplementary Table S1). The median TTF durations of atezolizumab treatment as the first- and second- or subsequent-line ICIs were 2.12 (95% CI, 1.86–3.61) and 1.64 (95% CI, 1.46–2.36) months, respectively. The difference was statistically significant (*p* = 0.04, using the log-rank test) (Figure 2A). We also evaluated the TTF duration according to the PS of patients, PD-L1 expression, and age group, but no significant differences were found. Regarding PS, the TTF durations were 2.04 (95% CI, 1.82–2.57) and 1.07 (95% CI, 0.964–not available [NA]) months for patients with a PS of 0 or 1 and 2 or 3, respectively (*p* = 0.07, using the log-rank test). Regarding PD-L1 expression, the TTF durations were 1.91 (95% CI, 1.46–2.36), 2.07 (95% CI, 1.75–4.36), and 2.04 (95% CI, 1.11–NA) months for patients with PD-L1 TPS ≥ 50, PD-L1 TPS <49, and unknown PS-L1 TPS, respectively (*p* = 0.2, using the log-rank test). Regarding age, the TTF durations were 1.75 (95% CI, 1.11–5.86) and 2.04 (95% CI, 1.82–2.54) months for patients aged ≥ 75 and <75 years, respectively (*p* = 0.5, using the log-rank test) (Figure 2B–D). In addition, we evaluated treatment efficacy with respect to the EGFR mutation status (Figure 2E, Supplementary Figure S1). The median TTF durations for patients who were EGFR mutation-positive (*n* = 8) and EGFR mutation-negative (*n* = 50) were 1.93 (95% CI, 0.93–NA) and 2.00 (95% CI, 1.75–2.54) months, respectively; the results were not statistically significant (*p* = 0.8, using the log-rank test). However, among patients who were EGFR mutation-positive, those who had low PD-L1 levels (TPS <49, *n* = 5) had a longer TTF duration than those who had high PD-L1 levels (TPS ≥ 50, *n* = 2) (*p* = 0.008, using the log-rank test). The patients were divided according to the presence or absence of previous ICI treatments, the reason for the change from a previous ICI was PD or otherwise, and the best response to atezolizumab (Figure 3). In 27 patients (Figure 3A) treated with ICIs (anti-PD-1 treatment: nivolumab or pembrolizumab) as second or subsequent lines, 17 patients were treated with nivolumab and 10 with pembrolizumab. Among these, one patient received nivolumab followed by pembrolizumab, and subsequently received atezolizumab as the third-line treatment. Sixteen (59.3%) patients (Figure 3B) with previous ICI treatments discontinued owing to PD. In addition, six (22.2%) patients (Figure 3C) discontinued owing to AEs, and five (18.5%) (Figure 3D) discontinued for other reasons (e.g., personal wish). Six (37.5%) patients (Figure 3E) had PR or SD, eight (50.0%) (Figure 3F) experienced PD because of atezolizumab when the reason for the discontinuation of the previous ICI was PD, one (16.7%) (Figure 3G) experienced PR or SD, and two (33.3%) (Figure 3H) experienced PD when the reason for the discontinuation of the previous ICI was AEs. All five patients (Figure 3I) who discontinued the previous ICI for reasons other than PD or AES experienced PD with atezolizumab. However, these differences in response to atezolizumab were not statistically significant (*p* = 0.468).

### 3.4. AEs

The adverse event (AE) profiles associated with atezolizumab treatment are presented in Table 3. Of the 74 patients, 12 (16.2%) experienced an AE of any grade. Grade 3–5 AEs were observed in 8.2% of the patients: grade 3 AEs included peripheral neuropathy, hypothyroidism, and rash, and grade 4 AE included hepatitis. Two deaths related to treatment were due to the acute worsening of interstitial pneumonia (IP). One of these two patients had IP as a comorbidity and was aged <75 years, whereas the other patient had no respiratory comorbidities and was aged >75 years, with no statistical difference (odds ratio = 1.63, 95% CI, 0.27–9.71, *p* = 0.5495). The patients who experienced AEs were followed up without steroid administration. However, the two patients who experienced acute exacerbation of IP (one with hepatitis and one with neuropathy) were treated with steroids. Eleven (14.8%) patients with previous ICI treatment developed irAEs and did not develop recurrence with atezolizumab. Among these 11 patients, 2 (18.2%) developed new irAEs: neuropathy (grade not reported) and grade 4 hepatitis. One patient developed grade 1 hepatitis when treated with nivolumab as the first ICI and did not develop recurrence when treated with pembrolizumab as the second-line ICI and atezolizumab. When assessed according to age group, three and nine elderly and non-elderly patients, respectively, developed irAEs, with no significant difference (odds ratio = 1.04, 95% CI, 0.24–4.36, *p* = 0.9526, Table 3).

## 4. Discussion

In this retrospective, a multicenter study involving 74 patients with advanced NSCLC, we demonstrated the real-world efficacy and safety of atezolizumab. In the POPLAR and OAK studies, the OS and PFS durations were 13.8 months and 12.6 months for OS and 2.8 months and 2.7 months, for PFS, respectively [7,8]. In this study, the OS and TTF durations were 7.54 months and 2.00 months, respectively. The OS and TTF in this study were shorter than those reported in previous studies [7,8]. We believe that the reason for the short TTF durations is that atezolizumab is the first anti-PD-L1 antibody drug to be used worldwide. Therefore, it was used with the expectation of being effective in patients with a low PS and a high number of previous treatment lines. De Giglio et al. [12] recently reported that patients with advanced-stage NSCLC received immunotherapy during the last few months, which is possible in the present study. In the present study, we were able to observe patients for over 5 years, allowing us to identify patients who survived for more than 6 years. Hence, our results may represent the true OS of patients who received chemotherapy including ICI.

We analyzed several predictive factors for efficacy, such as age, ECOG PS, histological subtype, and number of treatment lines. However, no significant differences were found. According to a previous study [13], an ECOG PS of 0 was a good prognostic factor. However, a PS ≥ 2 was found to be a poor prognostic factor [14]. Therefore, our results may differ from those previously reported, which may be explained by the limited sample size. Our study included more patients with lung cancer with a PS ≥ 2 than expected [15], but these patients are often excluded from clinical trials. PS 2 is observed in patients with several disorders, and they are often treated with ICIs in real-world clinical practices [16]. Thus, more real-world clinical data on ICI administration to patients with PS 2 needs to be accumulated.

A previous study showed that liver metastasis may be a predictor of poor ICI efficacy [17], and our data showed a similar tendency. This tendency has also been observed at other metastatic sites. However, we did not find a significant difference between patients with and without metastasis. Therefore, in clinical practice, avoiding ICIs based on the presence of metastasis may be unnecessary.

The median TTF durations of patients treated with atezolizumab as a first-line ICI and those treated with atezolizumab as the second- or subsequent-line ICIs were significantly different. The efficacy rates of ICI retreatment [18] and atezolizumab retreatment in patients who develop acquired resistance to anti-PD-1 antibodies [13] have been previously reported. However, our data showed that atezolizumab may not have an additive effect on survival in patients previously treated with anti-PD-1 antibodies. The therapeutic effect may be enhanced if atezolizumab is administered as early as possible. Anti-PD-L1 antibodies may be less effective in patients previously treated with anti-PD-1 antibodies, such as nivolumab and pembrolizumab [19]. Thus, the efficacy of ICI retreatment was not confirmed. However, when the analysis was restricted to those treated with previous ICIs, the effect did not change regardless of whether the previous ICI led to PD. Thus, our data did not provide any evidence to support avoiding atezolizumab simply because prior treatment with ICI resulted in PD.

No significant differences were noted in the incidence or severity of irAEs and TTF duration between elderly and younger patients. Although the number of cases is limited, these findings indicate that atezolizumab monotherapy may be safely and effectively utilized in elderly patients, including those who are often excluded from clinical trials due to comorbidities or reduced physiological reserve. Elderly patients often cannot participate in clinical studies, resulting in a scarcity of evidence-based treatment strategies for this age group. However, our data contributes to the growing evidence that ICIs, including atezolizumab, demonstrate comparable efficacy and safety in both elderly and younger patients. The efficacy of ICI use in elderly patients has been discussed previously [20,21], and several studies have indicated that the outcomes in elderly patients are not inferior to those observed in younger populations [22,23]. Our findings align with this perspective and support the potential of atezolizumab as a viable treatment option for elderly patients, helping to address the clinical needs of this vulnerable demographic. By demonstrating the comparable safety and tolerability of atezolizumab in elderly patients, this study highlights the importance of expanding the availability of ICIs for elderly individuals and conducting further research.

No significant differences were observed between patients with an EGFR mutation-positive and -negative status. However, when the analysis was limited to patients who were EGFR mutation-positive, those with a low PD-L1 TPS had a significantly longer TTF than those with a high PD-L1 TPS. In a previous study, among patients who were EGFR mutation-positive, ICIs were more effective in those with high PD-L1 TPS [24]. The reasons behind the difference in these results remain unclear. The small number of cases in this study was a potential contributing factor.

Looking at Figure 3, more patients had previously received nivolumab than pembrolizumab as an ICI treatment. This is likely because nivolumab was approved earlier for use in unresectable or recurrent non-small cell lung cancer (NSCLC), making it more commonly administered prior to pembrolizumab.

Regarding safety, no patients in this study who had developed irAEs developed a recurrence of their previous irAEs, and two patients developed new mild irAEs. Some patients who developed irAEs with the first ICI developed recurrences after ICI retreatment, and most recurrent and new irAEs due to the second ICI were mild [15,25]. Our data were consistent with the mildness of new irAEs, but not with the recurrence of irAEs. The incidence rate of irAEs of any grade reported in a previous clinical trial was 50–80% [26], and the incidence rate in this study was 12.2%, which is lower than that previously reported. Therefore, the reasons for the lack of recurrent irAEs may be the overall low incidence of irAEs and the strict selection of patients to avoid the recurrence of irAEs because there was no safety information about the recurrence of irAEs. One patient each with IP as an irAE in the younger and elderly group died.

In this study, the observation period was set to a maximum of six years, revealing that 6.7% of patients survived for more than five years. Although this result is lower compared to the four-year survival rates reported for the atezolizumab group in the POPLAR trial (14.8%) and the OAK trial (15.5%) [4], it is likely due to the inclusion of real-world data without strict patient selection criteria. These rates represent four-year survival data, highlighting the need for further long-term observational studies. The shorter survival outcome observed in this study could be attributed to the inclusion of a certain proportion of patients with poor performance status (PS).

The major limitation of this retrospective study is the small sample size. Owing to the small sample size, some differences may not have been apparent. Further accumulation of cases with longer follow-up periods, including elderly patients, is required. In addition, this was an observational study. Therefore, we were unable to establish a control group. Furthermore, the multicenter nature of this study made it challenging to unify the evaluations of recurrence and side effects; this may have affected the TTF duration and number of irAEs.

## 5. Conclusions

This retrospective analysis of 74 patients with advanced NSCLC demonstrated the efficacy and safety of atezolizumab monotherapy. Our findings suggest that atezolizumab may be effective when administered as early as possible and that its safety profile is also well-suited for elderly patients.

## Figures and Tables

**Table 1:** Baseline characteristics, stratification factors, and previous therapy (*n* = 74).

	Characteristic	(*n*/%)	Characteristic	(*n*/%)
**Age**			Genetic mutation	
	Median	70	EGFR	9 (12.2)
	Range	45-85	ALK	0
	Age	18 (24.3)	None	65 (87.8)
**Sex**			CNS metastasis	
	Male	56 (75.7)	Yes	10 (13.5)
	Female	18 (24.3)		64 (86.5)
**Disease stage**		No	Smoking status	
	IIA	1 (1.3)	Current or ex-smoker	57 (77.0)
	IIB	1 (1.3)	Nonsmorker	17 (23.0)
	IIIA	3 (4.1)	Treatment line	
	IIIB	5 (6.8)	Second line	29 (39.2)
	IVA	32 (43.2)	Third line or more	45 (60.8)
	IVB	17 (23.0)	Previous radiotherapy	
**Post Operative reccurence**		15 (20.3)	Yes	25 (33.8)
**ECOG PS**			No	49 (66.2)
	0	38 (51.3)	Previous ICI	
	1	31 (41.9)	Yes	27 (36.5)
	2	3 (4.1)	No	47 (63.5)
	3	2 (2.7)		
**Histologic type**				
	ADC	56 (75.7)		
	SCC	6 (8.1)		
	NSCLC, NOS	8 (10.8)		
	ASC	3 (4.0)		
	Pleomorphic	1 (1.4)		

Abbreviations: ADC, adenocarcinoma; ASC, adenosquamous carcinoma; CNS, central nervous system; ECOG, Eastern Cooperative Oncology Group; ICI, immune checkpoint inhibitor; NA, not available; NOS, not otherwise specified; NSCLC, non-small cell lung cancer; PS, performance status; SCC, squamous cell carcinoma.

**Table 2:** Relationship between patient clinical characteristics and treatment responses.

		Response				
Characteristic	Patients (*n*= 74)	PR or SD	PD	NA	OR (95% CI)	*p* Value
age. y						0.590
<75	56	12 (21.4)	33 (58.9)	11 (19.7)	1 (REF)	
≥75	18	5 (27.8)	7 (38.9)	6 (33.3)	1.46 (0.35-5.79)	
Sex						0.640
Male	56	13 (23.2)	28 (50.0)	15 (26.8)	1.48 (0.28-8.06)	
Female	18	4 (22.2)	12 (66.7)	2 (11.1)	1 (REF)	
ECOG PS						0.392
0–1	69	21 (30.4)	36 (52.2)	12 (17.4)	1 (REF)	
2–3	5	1 (20.0)	3 (60.0)	1 (20.0)	0.32 (0.01-3.44)	
Histologic type						0.590
ADC	56	13 (23.2)	29 (51.8)	14 (25.0)	1(REF)	
SCC	6	1 (16.7)	5 (83.3)	0 (0.0)	0.80 (0.33-3.31)	
Other	12	3 (25.0)	6 (50.0)	3 (25.0)		
EGFR mutation						0.412
No	65	16 (24.6)	35 (53.8)	14 (21.5)	1 (REF)	
Yes	9	1 (11.1)	5 (55.6)	3 (33.3)	0.38 (0.03-3.31)	
Smoking status						0.397
Nonsmoker	17	4 (23.5)	11 (64.7)	2 (11.8)	1 (REF)	
Current or ex-smoker	57	18 (31.6)	28 (49.1)	11 (19.3)	2.10 (0.39-13.1)	
CNS metastasis						0.285
Yes	10	0 (0.0)	4 (40.0)	6 (60.0)	1 (REF)	
No	64	17 (25.6)	36 (56.3)	11 (17.1)	2.98 (0.39-24.5)	
Treatment line						0.841
Second line	29	8 (27.6)	14 (48.3)	7 (24.1)	1 (REF)	
Third or more	45	14 (31.1)	25 (55.6)	6 (13.3)	1.07 (0.57-2.01)	
Previous radiation						0.242
No	49	12 (24.5)	25 (51.0)	12 (24.5)	1 (REF)	
Yes	25	10 (40.0)	14 (56.0)	1 (4.0)	2.20 (0.61-8.89)	
PD-L1 TPS						0.473
>50	17	1 (5.9)	14 (82.4)	2 (11.7)	0.73 (0.29-1.68)	
1–49	18	5 (27.8)	9 (50.0)	4 (22.2)	1(REF)	
0	27	8 (29.6)	13 (48.2)	6 (22.2)		
NA	12	3 (25.0)	4 (33.3)	5 (41.7)		

Abbreviations: ADC, adenocarcinoma; CI, confidence interval; CNS, central nervous system; ECOG, Eastern Cooperative Oncology Group; ICI, immune checkpoint inhibitor; NA, not available; OR, odds ratio; PD, progressive disease; PR, partial response; PS, performance status; REF, reference; SCC, squamous cell carcinoma; SD, stable disease.

**Table 3:** Treatment-related adverse events and the relationship between age group and adverse events.

Grade *n* (%)					Age Group (*n*)	
Event	Any	3 or 4	5	Steroid Use	<75 (*n* = 9; 16.1%)	75≤ (*n* = 3; 16.7%)
Hepatitis	1 (1.3)	1 (1.3)	0 (0.0)	1 (1.3)	1	
Hypothyroidism	1 (1.3)	1 (1.3)	0 (0.0)	0 (0)		1
Interstitial pneumonia	3 (4.1)	0 (0.0)	2 (2.7)	2 (2.7)	2	1
Neuropathy	2 (2.7)	1 (1.3)	0 (0.0)	1 (1.3)	1	1
Pericarditis	1 (1.3)	0 (0.0)	0 (0.0)	0 (0.0)	1	
Rash	4 (5.4)	1 (1.3)	0 (0.0)	0 (0.0)	4	

## Data Availability

Data are available from the authors upon reasonable request.

## Supplementary Materials

The following supporting information can be downloaded at: https://www.mdpi.com/article/10.3390/jcm13247815/s1, Table S1: Relationship between treatment response and metastasis; Figure S1: Kaplan–Meier plot for patients with an EGFR mutation positive-status and with PD-L1 TPS < 49 and PD-L1 TPS ≥ 50.

## Abbreviations

AEs	adverse events
ALK	anaplastic lymphoma kinase
CI	confidence interval
CNS	central nervous system
ECOG	Eastern Cooperative Oncology Group
EGFR	epidermal growth factor receptor
ICIs	immune checkpoint inhibitors
IP	interstitial pneumonitis
irAEs	immune-related adverse events
NA	not available
NSCLC	non-small cell lung cancer
ORR	objective response rate
OS	overall survival
PD	progressive disease
PD-1	programmed cell death protein 1
PD-L1	programmed death-ligand 1
TTF	time to treatment failure
PR	partial response
PS	performance status
SD	stable disease
TPS	tumor proportion score
