# Supplementary figures and images for: Real-World Efficacy and Safety of Atezolizumab for Advanced Non-Small Cell Lung Cancer in Japan: A Retrospective Multicenter Analysis
Source: J Clin Med. 2024 Dec 20;13(24):7815. doi: 10.3390/jcm13247815 (PMC11728080; doi:10.3390/jcm13247815)

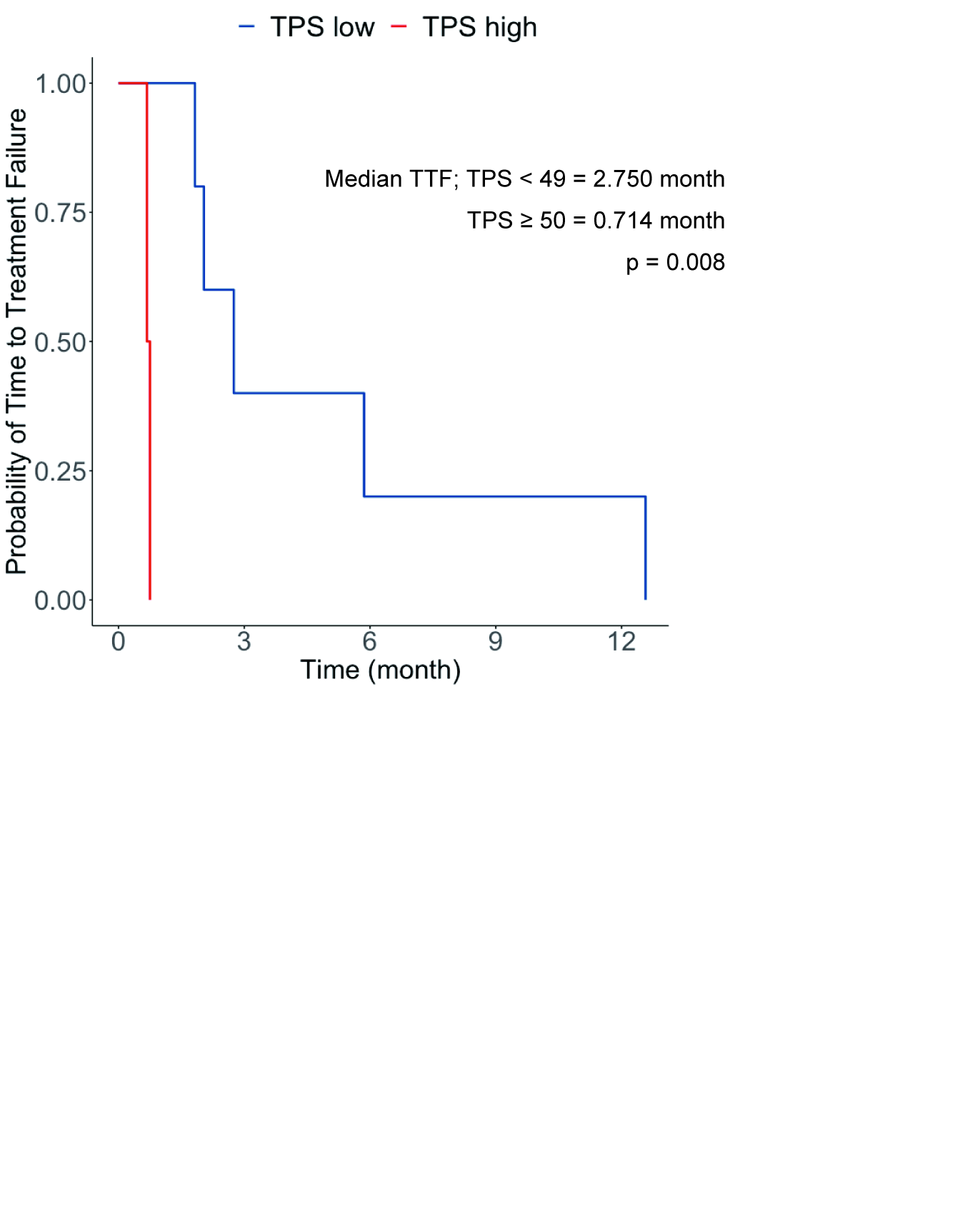

Supplement: Supplementary file 1 [file jcm-13-07815-s001.zip › supplementaryfigS1_241219.tif]
